# Understanding Patient–Provider Interaction, Treatment Acceptance, and Outcomes in Medically Unexplained Symptoms

**DOI:** 10.7759/cureus.32915

**Published:** 2022-12-25

**Authors:** Val Bellman, Tara Rava Zolnikov

**Affiliations:** 1 School of Behavioral Sciences, California Southern University, Chandler, USA; 2 Department of Psychiatry, University of Missouri Kansas City School of Medicine, Kansas City, USA; 3 Department of Community Health, National University, San Diego, USA

**Keywords:** sick building syndrome, healthcare barriers, chronic fatigue syndrome, idiopathic environmental intolerance, doctor patient relationship, exposure, toxicants, environment, chemical sensitivity, medically unexplained symptoms

## Abstract

Introduction: Medically unexplained symptoms (MUS) is an umbrella term used for chronic and often disabling health symptoms and conditions that remain unexplained after standard medical examinations, testing, and/or appropriate workup. Patients with MUS tend to receive little to no treatment but remain distressed, stigmatized, and disabled by symptoms and iatrogenic factors.

Methods: A qualitative phenomenological study was conducted to explore daily challenges and psychosocial and iatrogenic factors affecting the management of MUS.

Results: The analysis of the interviews revealed that MUS could cause significant distress to patients, impairing their functioning and leading to permanent disability. Conventional healthcare cannot meet the medical needs of these patients and might be a potential source of harm to them. It should be noted that confirmation of conditions associated with clinically significant psychiatric premorbidity was not provided.

Conclusion: Inconsistent diagnostic criteria, lack of proper training and research, diagnostic overshadowing, and implicit bias in healthcare professionals can lead to negative patient outcomes and the overuse of alternative or non-evidence-based services. Guidance, practice-based improvement ideas, and suggestions specific to improving patient-provider relationships can be applied to generate positive health effects.

## Introduction

Medically unexplained symptoms (MUS) describe symptoms that cannot be supported by an official medical diagnosis. Approximately 15-30% of primary care visits involve MUS, with approximately 10.5% of individuals experiencing at least one such symptom within a year [1]. MUS also include conditions like idiopathic environmental intolerance (IEI), which refers to a group of poorly understood medical conditions characterized by various physical symptoms that occur in response to environmental triggers [2]. Other environmental sensitivities produce symptoms that overlap with IEI, including chronic fatigue syndrome (CFS), IEI attributed to electromagnetic fields (IEI-EMF), and sick building syndrome (SBS). Moreover, exposure to industrial toxicants (heavy metals, pesticides, solvents) can lead to a myriad of vague or subtle presentations, often described as idiopathic or medically unexplained [3]. These symptoms are often not recognized as a specific medical condition, although they can cause significant clinical distress, difficulty in work or social activities, or adverse effects on quality of life. 

MUS are often caused by a combination of genetic and environmental factors that affect a patient’s presentation and severity of symptoms. Research has suggested that a biophysiological explanation can be found for only 26% of the 10 most common symptoms presenting in primary care, while industrial or environmental factors may also contribute to these presentations [4]. Historically, over two decades ago, a collaborative working group concluded that environmental factors play a significant role in the manifestation and progression of MUS [5]. The unknown nature of these symptoms can be the result of limited medical knowledge or technology or of the subjectivity of such complaints.

Because little to no research has been conducted on this issue from a person-experience standpoint, a qualitative phenomenological study was performed to understand the experiences of individuals with MUS, multiple chemical sensitivity (MCS), or chronic “functional” conditions after receiving a diagnosis while being treated by their healthcare providers. The primary goal of the study was to explore the unspoken nuances of healthcare delivery among patients with these chronic conditions. Psychosocial factors, including participants’ personal experiences and outcomes related to access and use of evidence-based help and quality of life outcomes, were also assessed. The results were expected to represent a significant step toward understanding patients’ perspectives, assessing healthcare barriers, and helping providers anticipate potential negative therapeutic outcomes as a result of poor patient-physician relationships. The overall aim was to provide a more comprehensive approach to understanding MUS and improving communication between patients, practitioners, and advocacy arenas by providing firsthand evidence about a phenomenon, participants’ experiences, and struggling to meet basic healthcare needs.

## Materials and methods

**Figure 1:**
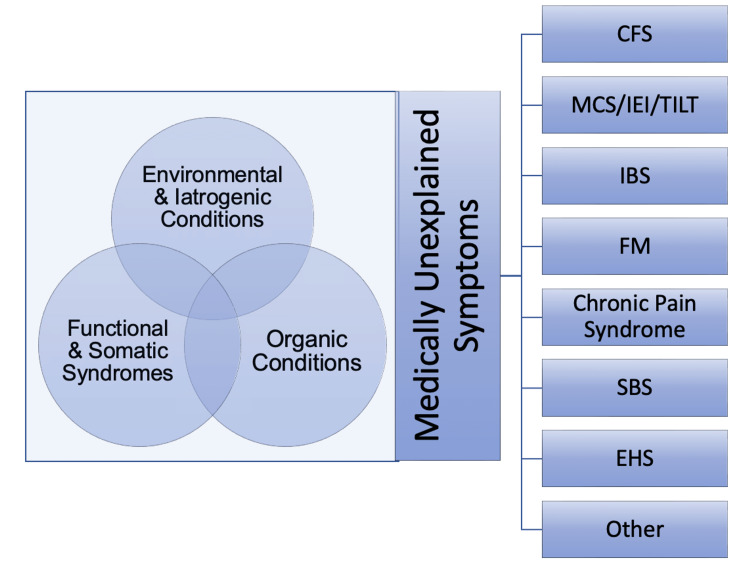
Medically unexplained symptoms and other conditions CFS: chronic fatigue syndrome; MCS: multiple chemical sensitivity; IEI: idiopathic environmental intolerance; TILT: toxicant‐induced loss of tolerance; IBS:  irritable bowel syndrome; FM: fibromyalgia; SBS: sick building syndrome; EHS: electromagnetic hypersensitivity

A qualitative research methodology was utilized in this study to understand and explore participants’ experiences with both certain providers and healthcare delivery in general [6]. This research design was employed in this study to investigate a distinct phenomenon characterized by a lack of detailed preliminary data on the topic. While other methodologies are deployed to generate opinions and produce a generalization through hypotheses, phenomenology seeks to evaluate participants’ lived experiences associated with many influences (e.g., previous exposure to toxicants, healthcare-related issues, low socioeconomic status, culture, personal history, and societal views) [7]. Finally, this methodology permitted a focus on contextual meaning through the situational knowledge of those being researched and conveyed the needs of individuals with MUS, MCS, and similar conditions who are commonly perceived or regarded as hypochondriacs, “attention seekers,” or high utilizers [8-9]. Figure 1 outlines the most common MUS and relevant conditions. 

The target population included various individuals who believe they are sensitive to environmental toxicants or are suffering from medically unexplained, recurrent, and nonspecific psychological and physical symptoms. Specifically, a convenience sample of individuals with self-reported histories of IEI/MCS, similar conditions, and MUS was drawn from internet forums and social media, mainly Facebook (Meta Platforms, Inc., Menlo Park, California, United States), between March 1, 2021, and May 31, 2021. Table 1 summarizes the inclusion and exclusion criteria used during the screening process.

**Table 1:** Study inclusion and exclusion criteria

Inclusion criteria	Exclusion criteria
Individuals above 18 years of age (regardless of gender, race/ethnicity, or socioeconomic status)	Individuals under 18 years of age and over 75 years of age
Voluntary willingness to participate in the study	Individuals with organic brain diseases (e.g., stroke, tumor)
Individuals located in the United States or other English-speaking countries and/or able to communicate in English	Individuals with present or past psychotic disorders and/or substance use disorder
Individuals diagnosed and/or having a self-reported history of hypersensitivity to environmental factors, MUS, and other toxicant-induced disorders	Presence of a well-defined (diagnosed and/or recorded) somatic disease that could account for the reported bodily complaints (e.g., traumas, autoimmune conditions such as lupus, scleroderma, systemic vasculitis, infections)
Individuals with a self-reported history or presence of any psychological disturbances and/or symptoms related to their conditions and healthcare delivery issues	Individuals with unstable formal psychiatric disorder and/or unable to give informed consent
Individuals exhibiting or reporting any changes in daily routine, impairment in socio-occupational functioning, and challenges with healthcare delivery/unmet healthcare needs	Individuals who could not abstain from any mood-altering substances (with the exception of medications/supplements prescribed by their provider) for at least eight hours before the interview session

For the purposes of this study, passive and active recruiting were used to obtain a large population and to make selections based on personal information that was already available to determine the eligibility of potential participants. Participants were screened, selected, and provided with an informed consent form via DocuSign (DocuSign, Inc., San Francisco, California, United States) to inform them about the aim and objectives of this study. After discussing informed consent, a select number of participants who agreed to participate in the study process were evaluated via Zoom (Zoom Video Communications, Inc.San Jose, California, United States), Skype (Skype Technologies S.A.R.L, Luxembourg City, Luxembourg), and other available online platforms (messengers, emails) to maintain social distancing recommendations by the Centers for Disease Control and Prevention (CDC) concerning the coronavirus disease 2019 (COVID-19) pandemic. The semi-structured interviews were formulated as a guide to collect relevant data. The interview sessions lasted around 60 minutes; all responses were recorded and analyzed via manual coding, where themes emerged and presented themselves through repetition. The themes were then entered into a code spreadsheet, which was used to review all quotes related to the subject matter that directly correlated to answering the research questions. 

The researchers also established trustworthiness through credibility, multiple participant perspectives, peer debriefing and review, and field notes conducted during each session. Multiple participant perspectives were sought when participants from different countries, with multiple and possibly heterogeneous viewpoints, representations, and roles, were included in the study [7]. Peer review and debriefing were conducted before and after developing interview questions, reviewing participants’ responses, generating emerging and final categories/themes, and producing the final results of this study [10]. We also utilized a skilled moderator to ensure the validity of the data and overcome the researchers’ personal bias. Further, the validity of the research was supported by the respondents’ validation of the results. The reliability of the research was confirmed through the triangulation of sources, member-checking, and the use of comprehensive data, tables, specific software, and a comparison of the findings. The study protocol and ethics review were approved by the Institutional Review Board, California Southern University, Chandler, United States. The researchers also assigned an identifying code to each participant in order to ensure the confidentiality and anonymity of the research. Finally, Research Quality Review (RQR) guidelines were utilized to report the findings [10]. 

## Results

A total of 57 individuals, comprising adult men and women ranging in age from 34 to 75 years, initially agreed to participate in the study. Of these 57 participants, 15 were lost to follow-up (e.g., some of the participants became unreachable or disengaged from the study due to frustration and/or conflicts with the timing of the research process, while others withdrew as they found the study to trigger negative memories, emotions, and/or feelings). Of the remaining 42 participants, 36 were women and six were men, all fluent in the English language. Of these participants, 34 identified as Caucasian/White, three as African American/Black, three as Latin, and two as Asian American. The participants demonstrated a diverse range of symptoms and environmental exposures, including MCS, industrial chemical poisoning, heavy metal exposure, and various experiences with healthcare providers in four different healthcare systems: United States (31), Canada (5), Australia (3), and Russia (3). In terms of educational background, 14 participants had completed a master’s degree, 12 had completed a bachelor’s degree, and three had completed a doctoral degree. Additionally, seven participants had completed some college-level courses, and six had a high school diploma. None of the participants shared the same academic major or occupational background, which provided for diverse perspectives. Likewise, none of the participants had been formally diagnosed with a mental illness. 

The data analysis highlighted main themes, including participants’ negative experiences with healthcare providers, poor compliance and treatment avoidance, use of complementary providers and alternative treatment, and poor life quality. These were reviewed again and consolidated into 16 emergent themes. The following themes and ideas were employed to organize and explain the participants’ shared experiences with healthcare providers and treatments. 

Negative interactions with providers

Regardless of the time associated with the manifestation of toxicant-induced conditions, each participant shared a long-term (10+ years) history of health-related complaints specific to previous environmental exposure(s). The participants felt devalued while experiencing varying degrees of stigmatization or discrimination because of their illness. P.0825-21 stated:

The healthcare community has made it very clear that I have no value as a human.

The participants also acknowledged that healthcare-related stressors may indeed exacerbate physical symptoms, regardless of the type and/or duration of exposure. According to P. 0705-02:

Those people in medicine want an easy fix and there’s no easy fix for people like me and that frustrates them, and they take it out on us. It makes me feel even worse.

The participants emphasized that physicians tended to minimize or disregard disabling and/or dangerous symptoms described by patients. For example, P.0215-13 stated: 

Every time I go to the doctor, I sit on the examination table and mentally prepare myself to be disbelieved. Some doctors expressed a “my-way-or-the-highway” approach to treating me, meaning I had to take whatever meds they said without question nor discussion. Otherwise, they would not treat me.

Of those MUS/ED participants with a reported physician gender preference, most female participants preferred female providers as they communicated better and spent more time with patients. Male providers were described as appearing to be biased, which resulted in poor treatment and labeling. P. 0138-35 described how she was considered a “difficult woman” because she did not agree with the diagnosis:

The worst (part) is when a man is helping a woman… (and) that the woman is imagining… her symptoms.

Even though most states have laws that protect the Lesbian, gay, bisexual, and transgender (LGBTQ) community against differential treatment, at least one participant (P. 0935-34) experienced discrimination because of their “perceived” sexual orientation or gender identity:

I dealt with the doctor who treated me badly because he thought I was gay.

The participants emphasized the connection between the progression of symptoms and the physicians’ attitudes. According to (P. 0414-27):

I get depressed and very anxious with doctors when I can’t get through to them the seriousness of my illness. That makes my symptoms worse and I can’t function as before.

The participants stated that physicians attributed MUS to a myriad of problems, including psychological distress, intolerance of otherwise benign symptoms, social problems, or personal gain from sickness. Thirty participants experienced the phenomenon of pathologizing, which occurred when their provider made an assumption based on a preconceived notion or idea. In these scenarios, the patients perceived their physician to be judgmental and stigmatizing when evaluating their symptoms and concerns. According to participant P.1247-14:

Doctors don’t know about this condition…or they believe that this condition is, like, ‘all in your head.

Reportedly, some healthcare professionals attributed physical complaints (e.g., pain, tachycardia, hypertension, shortness of breath, seizures) to manifestations associated with a pre-existing mental health disorder. P. 0705-02 said:

I’ve had providers who say: ‘I don’t know what to do for you, It’s likely psychogenic.'

Poor compliance and treatment avoidance

The participants explained how expectations of medical services or avoiding medical services were common among them. The avoidance of medical services was associated with previous extensive testing and diagnostic processes undergone that had resulted in little to no diagnostic success. Because MUS/IEI are not within the range of a traditional medical diagnosis, addressing and treating them is not well known or understood by medical professionals. According to P. 1247-14:

(Most doctors) admit that they don’t know much about it.

These patient-physician relationships were often associated with decreased self-reported psychosocial well-being and greater physical distress: *“It’s very mentally challenging to live with…”* (P. 0739-28). The avoidance of medical care altogether was an anticipated effect of this situation. According to P. 0705-02:

I will go out of my way to avoid seeking medical care. I have had episodes of tonsillitis completely unrelated to chemical injuries that I had let go untreated because I didn’t want to go (to the doctor’s office).

In interviews with MUS/MCS sufferers, it was found that patients believed that most Western medical professionals had little to no knowledge of odor sensitivity and resulting symptoms. Experiences included difficulty navigating MUS symptoms and treatment. For example, P. 0110-37, who had a history of pesticide poisoning, explained: 

The specialist agreed to prescribe me an antidote with some powerful absorbing properties, but I found its formulation to be suspicious…the fact that I assumed the physician to be not qualified enough also played a role in not taking the prescribed medicine. 

Additional healthcare challenges 

Other “external” factors, such as insurance policy and local healthcare standards, negatively affected the patient-physician relationship, which resulted in physicians being unable to meet patients’ needs. Advocacy was wanted and needed. P. 0825-21 said:

Their job is to support the patient…they have a voice within the medical system that’s bigger than us and they should be using that voice to meet the patient’s needs.

Despite the legal and ethical regulations that physicians receive for patient care, they are seemingly ignored during patient interactions: According to P. 0119-15:

(The healthcare provider) began spraying (fragrant Lysol) around the entire waiting room while I was seated there, knowing I have asthma triggered by similar chemicals. (I asked them to stop, but) they refused to stop using Lysol and told me to go to my primary care provider to manage my ongoing care. I could not find another specialist in our area.

Patients also discussed symptoms first with general practitioners or primary care physicians. Typically, the nature of these interactions did not provide adequate time to explore symptoms, history, and potential solutions for a complex condition, such as an MCS. Participants reaffirmed that these practitioners needed to be educated on cultural competence as it related to individuals with chronic and disabling symptoms, in general, and with MUS, in particular. As a result, these patients were disappointed and dissatisfied after their initial interactions. P. 1507-30 said: 

I was distraught (with) the first physician I tried to seek help from, who would not even come into the room…this person was unwilling to come face to face with me to even discuss the situation.

Utilization of alternative health services

The participants’ responses demonstrated a preference for alternative medicine among patients with MUS/ED because of inadequate treatment through Western medicine. They also faced difficulties accessing evidence-based information and instead utilized services offered by unaccredited organizations and unlicensed medical facilities. Complementary practitioners (e.g., chiropractors, massage therapists, acupuncturists) were more knowledgeable, sympathetic, and respectful. According to P. 1247-14:

*At least (complementary practitioners) try to understand it…they take the most detailed histories ever...it’s really impressive.* 

The participants felt their concerns were being attended to, which made complementary alternative providers appear to be more educated regarding difficult diagnoses. P. 0252-39 said:

I feel like actually, listen to me…and I do think they’re better educated. 

Some participants emphasized that they felt more comfortable calling out-of-state complementary providers and traveling significant distances rather than considering Western medicine physicians, practices, and treatments. According to P. 0339-43:

I will need to travel two hours to see my holistic dentist in Halifax to have my two broken teeth repaired.

Poor quality of life outcomes 

The participants experienced negative outcomes and a poorer quality of life resulting from their MUS. Difficulties occurred financially within occupational settings and other areas. Alongside physical health, these situations also significantly affected mental health. According to P. 0834-22: 

This illness (MCS) is destroying my life…I’ve sold everything to fight this terrible condition trying to cure it. I was homeless for about three years because of it…I have had to work and sleep in my car because I could not breathe in the house. It was eye-opening when I was kicked out of a parking lot and all I wanted to do was get a couple of hours of sleep.

Participants with chronic disabling symptoms who had previously qualified for a job had to apply for reasonable accommodations in their workplace, such as a modification of the attendance policy. P. 0219-19 said:

I have had to apply for reasonable accommodations to seek diagnosis and treatment, as well as to work in a safe environment. 

Other participants reported that healthcare providers refused to supply paperwork that was needed to get a reasonable accommodation at work or for social security disability benefits or other public aid. Without these supporting documents, the participants were unable to receive the assistance needed to accommodate their lives. According to P.0810-49:

I was denied disability in 2019. My lawyer explained to the judge that in his 35 years he had never come across a case like mine. The determination letter stated there were thousands of jobs nationwide that I could do with these restrictions.

The participants who were unable to receive accommodation were forced into working conditions that adversely affected their health. According to P. 0378-45: 

I have had asthma for a long time, and it was well controlled until two years ago when I started working in a factory manufacturing car batteries. Now I have trouble breathing even with asthma medications. I took the job because of the pay. (Now) I get sick every time I go to work, but I can’t quit. I have to work and support my family.

The participants with chronic MUS described how their conditions were accompanied by mental health effects, such as neuroticism, chronic stress, anxiety, and depression. Many participants indicated that exposure to certain toxicants was psychologically traumatizing. None of them mentioned any similar behavioral or psychological problems prior to exposure. The MUS affected their lives, including but not limited to relationship and occupational issues. P. 1028-20 said: 

I do not travel, I have few friends, I try not to bring others into my home, no vacations, no motels…People lose their friends and many times their families…it can still bring me to tears.

Adverse mental health frequently resulted from lack of treatment or accommodations and continued suffering from symptoms. The participants described feeling anger. The participants expressed feeling despair and hopelessness. According to P. 0110-37:

*I’ve lost my life. 8+ years, gone…. I feel like a rat in a cage…*I* am at the end of my rope… I have tried more things than most can even believe*.

Lack of help, empathy, and solutions lead to sadness and powerlessness. P. 1137-11 said:

It seems like an endless story.

The desire to be seen and heard while removing the invisibility enveloping participants was frequently mentioned. This situation was highlighted during the COVID-19 pandemic, which subjected everyone in the world to feelings of isolation. According to P. 0908-25:

Now I see everyone, mostly everyone wearing a mask. I feel like yelling “Welcome to my world, how does it feel?” This lockdown hasn’t really affected me since I already live a life of quarantine.

Stress and anxiety were also mentioned, as the participants described what it was like to be *“…living in never-ending stress and suffering from this illness”* (P. 0378-45).

## Discussion

The measure of MUS severity is based on the number of self-reported symptoms; moreover, patient-reported severity is often used to classify individuals as healthy or disabled. Given the nature of this population, it was difficult to formally examine the internal consistency, test reliability, and predictive validity of reported symptoms and case-specific presentations. Some authors have also claimed that validity has not been established for any MUS across all medical specialties [11]. Table 2 shows the range of diagnostic labels given by different specialists to patients with MUS [12]. 

**Table 2:** Medically unexplained symptoms (MUS) and diagnostic labels across medical specialties.

Medical specialty	Unexplained symptoms	Diagnostic label
Neurology	Weakness, seizures, sensory disturbances, and abnormal movements	Functional neurological symptom disorder, conversion disorder, dissociative disorder
Psychiatry	Somatic symptoms and abnormal thoughts, feelings, and behaviors related to symptoms	Somatic symptom disorder, somatization disorder, dissociative disorder
Gastroenterology	Abdominal pain, diarrhea, bloating, constipation, excessive flatulence	Irritable bowel syndrome, non-ulcer dyspepsia
Cardiology	Chest pain, palpitations, fainting	Atypical chest pain, *acute coronary syndrome* (*ACS*)
Rheumatology	Joint pain, fatigue, headaches, sleep disturbance	Fibromyalgia, chronic pain syndrome
Infectious diseases	Fatigue, headaches, poor concentration, joint pain	Chronic fatigue syndrome (myalgic encephalomyelitis)
Dentistry	Facial pain, headaches, tinnitus	Atypical facial pain, temporomandibular joint disorder
Allergology	Fatigue, burning eyes, breathlessness, poor concentration, weakness, dizziness, lump in throat, breathing problem	Food/drug allergies, multiple chemical sensitivity, globus syndrome
Pulmonology	Breathlessness, rapid breathing	Hyperventilation syndrome, cough hypersensitivity syndrome, upper airway cough syndrome (UACS)
Obstetrics and gynecology	Pelvic pain, pain during sex, dysmenorrhea, painful urination, urinary retention	Chronic pelvic pain

It is known that diagnosis is the mainstay of medical care and, without it, it is difficult for medical professionals to provide effective care. This situation makes MUS extremely sensitive and difficult to treat within a standard healthcare setting. That said, patients still desire the same standard of care from healthcare providers, one which offers a well-defined description of symptoms and an explanation of the origin of the disease, reasonable explanations, and subsequent treatment [13].

Being diagnosed with MUS is not uncommon; many symptoms cannot immediately be explained as a recognized medical condition. A comprehensive psychiatric evaluation may be needed to rule out emotional, behavioral, or developmental disorders in this population. Because of vague manifestations, providers frequently misdiagnose MUS as a purely psychiatric disorder [14-15]. Many patients who have reported chronic physical symptoms were found to have associated symptoms of anxiety or depression, although these did not meet the criteria for a formal psychiatric diagnosis [16]. Therefore, treating the associated affective symptoms may help to relieve the physical symptoms experienced by patients [17]. Although MUS and MCS/IEI are frequently labeled as “functional” or “psychogenic” disorders, our results suggest that these conditions also occur in patients without obvious psychopathology, arguing against a causal role of psychiatric factors in MUS. 

The difficulty surrounding MUS is comprehensive, as supported by the findings in this study. There were numerous negative interactions with healthcare providers, which resulted in both poor compliance with treatment and increased use of complementary alternative treatments by alternative providers. MUS led to poor quality of life outcomes, which included stress and sadness (e.g., anxiety and depression) due to problems in relationships, work, and normal daily life settings.

**Figure 2:**
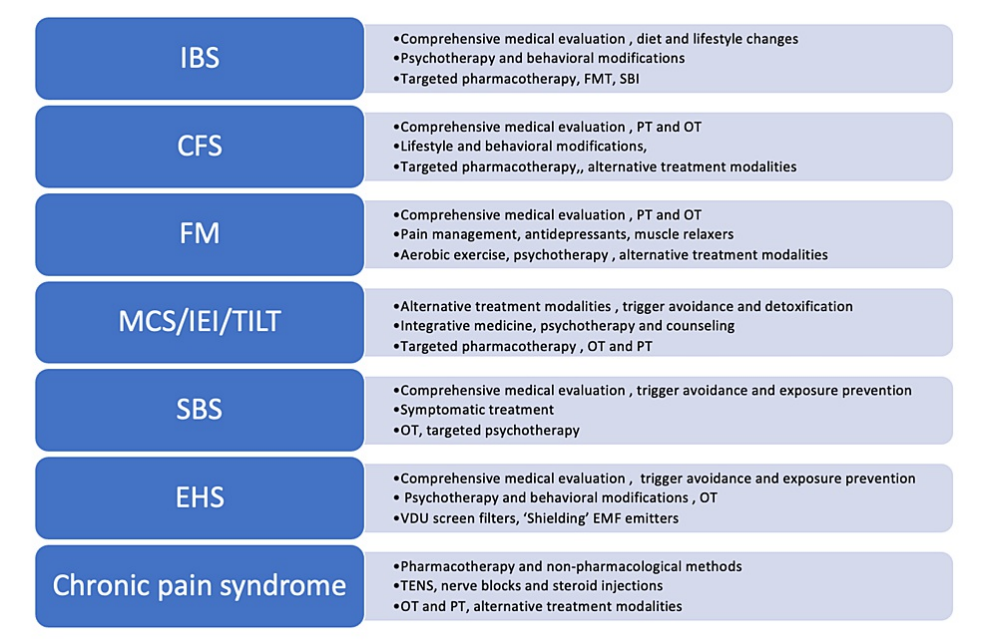
Overview of clinical practice guidelines in MUS CFS: chronic fatigue syndrome; MCS: multiple chemical sensitivity; IEI: idiopathic environmental intolerance; TILT: toxicant‐induced loss of tolerance; IBS:  irritable bowel syndrome; FM: fibromyalgia; SBS: sick building syndrome; EHS: electromagnetic hypersensitivity; FMT: fecal microbiota transplantation; SBI: serum-derived bovine immunoglobulin; TENS: transcutaneous electrical nerve stimulation; PT: physical therapy; OT: occupational therapy; MUS: medically unexplained symptoms

More importantly, this study suggests that people with chronic disabilities, like those with MUS/MCS, may be in danger of being denied healthcare by not having a voice in a healthcare setting. Although some recommendations regarding MUS and MCS/IEI patients have been published, many of these guidelines have generated controversy and have left patients and clinicians confused and distrustful [18-19]. Figure 2 provides an updated consensus on both the non-pharmacological and pharmacological treatments of MUS.

Despite the growing number of guidelines, many MUS patients cannot be managed by standardized protocols because of clinically valid reasons. Providers also feel that data about what to recommend are often lacking and misleading. Moreover, some recommendations are influenced by the experiences and misconceptions of policymakers. Finally, official guidelines for MUS are often focused on cost-benefit analysis, not patients’ needs [20]. The effectiveness of conventional physical treatment by primary care providers is currently limited, while the efficacy of behavioral modifications is well established [21]. Besides the suggested benefits of psychotherapy, the search for disease-modifying treatment modalities has not been successful. The role of pharmacotherapy is limited to symptomatic management in a minority of patients [22]. Finally, many individuals with MUS may improve without any specific treatment, particularly when their provider gives an explanation for symptoms that makes sense, removes any blame from the patient, and generates ideas about how to manage their symptoms [23]. 

Another important hindrance in providing equality in care is that the condition is often not recognized as a “valid” form of disability. Patients with MUS/MCS have significant problems with providing formal evidence since some of their symptoms are not objective, the conditions are not “recognized,” and they cannot be formally treated with standard medication or effectively tested at the primary care level [24-26]. Because research on the management of MUS and MCS/IEI is still limited and not well funded, it is difficult to generate validating items that could be used for disability needs or accommodations [27-28]. This study revealed that patients with MUS and similar conditions may become partially or totally disabled for several years or for the entirety of their life. Although there is no formal definition regarding disability in patients with MUS/MCS, it is clear that these symptoms negatively affect social, occupational, and family-related aspects of their lives. For example, these patients can experience difficulty in filing disability paperwork or insurance claims. The Social Security Administration (SSA) lists 14 specific types of adult impairments that automatically qualify affected individuals for Social Security Disability Insurance or Supplemental Security Income. Unfortunately, MUS are not on this list of impairments, although patients may still qualify if they can demonstrate “medically determinable impairment” [29]. Fortunately, the United States Department of Housing and Urban Development and the SSA have recognized IEI as a disabling condition and at least eight states in the United States have also formally supported MCS-related activities (e.g., MCS awareness month) [30]. Table 3 summarizes some examples of policy- or management-related ideas focused on equality for patients with MUS. 

**Table 3:** MCS/IEI management: general recommendations for providers and healthcare policymakers MCS: multiple chemical sensitivity; IEI: idiopathic environmental intolerance

Provider-specific adjustments	Hospital/system-based adjustments
Healthcare workers and/or providers must undergo MCS-focused training	Standardizing diagnostic criteria for MCS/IEI
Detailed history taking, patient-focused approach in managing complaints	Implementing a fragrance-free policy (no artificial scents, perfume) for staff, patients, and visitors [22] [24]
Patient’s constant involvement in healthcare decision making	Avoiding use of cleaning products that contain chemicals and/or synthetic fragrances (Hydrogen peroxide for disinfection as suggested by some authors [22])
Appropriate referrals if clinically necessary	Arranging a private room (decontaminated, cleaned, and properly ventilated) if possible (>6 hours before admission)
Behavioral health interventions, counseling, psychotherapy for symptomatic vs. functional improvement (if accepted by the patient)	Arranging a dedicated pre-entrance vestibule/room for staff where they can change their clothes
“Start low, go slow” approach in pharmacotherapy if clinically indicated	Ensuring the availability of “MCS kits” (shirts, powder-free vinyl or nitrile gloves, latex-free and phthalate-free oxygen tubes/masks) [22]
Avoiding replacing tolerated medications with generics	Marking all allergies, adverse drug reactions, and tolerated drugs in EMR/patient’s chart
Improving patient quality of life with feedback to providers about functional status	Activating MCS-related research in order to improve access to evidence-based information
No perfume policy for staff	Accepting MUS/IEI as a form of physical disability
Providers and staff should avoid smoke, perfumes, hair gel, or deodorants six hours before any planned interaction with chemically-sensitive individuals	Conducting research to influence federal/SSA policy changes
Recommend medical kit for MCS patients in daily life	Expanding health insurance coverage for patients with chronic physical and psychological problems in the context of MCS/IEI
Provider-conducted evaluation of prescribed medications, supplements, and medical devices (i.e., avoid colored tablets, avoid polypharmacy, assess non-active compounds, avoid plastic)	Standardizing alternative treatment modalities and proper credentialing of practitioners

Treatment avoidance and the use of alternative providers and treatments were highlighted in the results of this study. Treatments for MUS/IEI patients (e.g., creating a chemical-free living space and chemical avoidance, patient-centered care, guided self-help and specialist referrals, selective pharmacotherapy, and psychological interventions) mostly focus on reducing the severity of symptoms and reducing disability and associated distress. 

A viable and sustainable solution for this situation could be to encourage healthcare providers to demonstrate empathy toward MUS patients. Specifically, MUS patients, once validated the legitimacy of their disease has been recognized, are more satisfied, more likely to agree with their provider, and more likely to experience problem resolution [31]. In fact, legitimizing a patient’s symptoms is one of the most widely recommended strategies when treating patients with MUS [9]. Research has shown that physicians who discuss components of MUS and treatments in primary care settings promote better patient care and adherence to treatment [32]. To cater to a more general understanding of the disorder, providers should be well-versed and educated about these conditions. A foundational understanding of MUS can promote more efficient evaluation, treatment, and counseling of these patients [33]. 

Other changes need to occur within the “implicit” biases that contribute to disparities in healthcare delivery [34-35]. Some of these biases could be obscured by the fact that healthcare providers are deemed more knowledgeable than patients about disorders. Physicians may assume that patients with MUS and MCS/IEI are illiterate, uneducated, or even cognitively impaired. This is not the case; in fact, previous studies have suggested that chemical hypersensitivity is more common in highly educated populations [36], although individuals with MUS are usually less educated than the general population [23]. The results of this study suggest that participants attempted to seek treatment and be diagnosed with specific conditions, which is where providers could actively use and involve MUS/IEI patients in the medical decision-making process instead of disregarding the information that they may have about their disorder. Furthermore, previous studies have indicated that multi-component and interdisciplinary approaches are most helpful for patients with MUS, IEI, and similar conditions [19,37]. This type of relationship and interaction could improve compliance and treatment and lead to overall improved quality of life for these patients.

The results of this study should be viewed with caution due to the following limitations. First, there is little confirmatory evidence available to identify patients who are at risk of developing MUS or IEI based on the type or extent of their environmental exposure. Disability in patients with MUS depends on other subjective factors, like the frequency and intensity of symptoms. Another limitation of this study was the absence of well-defined diagnostic criteria, which may raise some legitimate questions regarding the validity of the results and the degree to which they can be generalized. Like many phenomenological studies, this qualitative research was mainly focused on having an experience-related understanding of the phenomenon, which is not meant to be used for generalizability or causality. A qualitative research methodology may be associated with some difficulties in understanding or confirming the basis of the phenomenon, collecting data, extensive data analysis and interpretation, and non-specific and/or clinically irrelevant answers given by the participants. This may underestimate the strength of the association between the number of complaints, clinical significance, and the degree of impairment. 

## Conclusions

Persistent MUS and conditions like IEI are associated with a variety of poor patient outcomes, including increased disability, poor quality of life, and unmet healthcare needs. Patients with MUS/MCS have significant problems providing formal evidence since some of their symptoms are not objective, their conditions are not “recognized,” and they cannot be formally treated with standard medication or effectively tested at the primary care level. This study has shown that patients with MUS often are not able to have their basic healthcare needs met and experience frustration with conventional medicine in general. Although the term MUS is widely used in clinical practice and professional literature, the results from this study also suggest that people with MUS are often not met with respect or empathy, which eventually leads to patients’ disengagement from evidence-based services.

Future research could review the use of a formal diagnosis for MUS. For example, patients could be given a specific diagnosis of a syndrome(s) that most accurately describes their main symptoms. This type of approach could significantly improve the physician-patient relationship and patients’ health outcomes and could potentially eliminate barriers to healthcare access. Other future research could focus on the development and implementation of more integrative treatment protocols to improve the diagnosis, treatment, and prevention of these conditions. A collaborative care model that integrates mental health services in the general practice setting would be an effective intervention in the treatment of MUS. This intervention should involve a stepped care approach, with the intensity of the intervention being proportional to the complexity of the patient’s problem.
